# Angiomyolipoma during pregnancy: A forgotten risk factor for rupture and massive haemorrhage - A case report

**DOI:** 10.51866/cr.399

**Published:** 2024-04-24

**Authors:** Khairil Amir Sayuti, Saiful Amri Mat Rasid, Mohd Shafie Abdullah, Siti Jusna Muhammad

**Affiliations:** 1 MBBCh, MMed (Radiology), Department of Radiology, School of Medical Sciences, Universiti Sains Malaysia, Kubang Kerian, Kota Bharu, Kelantan, Malaysia. Email: khairilamirsayuti@yahoo.com; 2 MBBCh, Department of Radiology, School of Medical Sciences, Universiti Sains Malaysia, Kubang Kerian, Kota Bharu, Kelantan, Malaysia.; 3 MD, MMed (Radiology), Department of Radiology, School of Medical Sciences, Universiti Sains Malaysia, Kubang Kerian, Kota Bharu, Kelantan, Malaysia.; 4 MD, MMed (Radiology), Department of Radiology, Hospital Serdang, Selangor, Malaysia.

**Keywords:** Angiomyolipoma, Pregnancy, Rupture, Retroperitoneal Haemorrhage

## Abstract

Benign renal lesions are relatively rare. Angiomyolipoma (AML) is the most commonly encountered benign renal lesion. One of the complications of AML is rupture, which results in retroperitoneal haemorrhage with a mortality rate of up to 20%. Pregnancy poses a major risk for the rupture of AML. This is attributed to its hormonal effect, which causes the tumour to grow rapidly during pregnancy. The possibility of AML rupture should be considered when encountering pregnant patients with hypovolemic shock but with normal initial obstetric ultrasound findings. We present a case of a pregnant patient who was admitted with hypovolemic shock and CT scan confirmed rupture of AML.

## Introduction

Angiomyolipoma (AML) is a rare benign lesion, affecting 0.2%–0.6% of the total population worldwide. The life-threatening complication of AML is rupture with retroperitoneal haemorrhage, which occurs in 15% of patients with AML. Pregnancy is an established risk factor for rupture due to its sensitivity to oestrogen and progesterone. Most non-pregnant patients with AML are asymptomatic, while the majority of pregnant patients with AML develop complications, most commonly rupture.

Embolisation and nephrectomy are the main interventions for the life-threatening complication of AML. The involvement of multiple disciplines such as family medicine, urology, obstetrics and gynaecology, paediatrics, intensive care and intervention radiology is crucial in deciding the treatment approach. Some factors to consider include maternal and foetal conditions, gestational age, availability of facilities and patients’ preferences for delivery.

Herein, we report the case of a young female patient in her 27th week of pregnancy who presented with abdominal pain that progressed rapidly into hypovolemic shock. Immediate computed tomography (CT) and urgent intervention were conducted to save this patient’s life.

## Case presentation

A 37-year-old Malay lady at 27 weeks of pregnancy with no significant medical history presented to the emergency department with severe abdominal pain. Due to normal initial obstetric ultrasound findings and stable vital signs, she was admitted to the ward for close observation.

After a few hours in the ward, her blood pressure suddenly dropped to 89/55 mmHg; her heart became tachycardic (143 beats per minute); her extremities became cold; and her haemoglobin level dropped from 10 g/dL to 7 g/dL. She was then urgently sent to the operating room for the possibility of abruptio placenta. Caesarean section was performed, and the foetus did not survive. The uterus was found to be normal. However, a left retroperitoneal haematoma was noted.

The surgical team was called to the operating room, and an exploratory laparotomy was then performed to find the source of bleeding. However, due to non-pulsatile, non-expanding haematoma, the surgeon decided to close first and requested an urgent CT to find the source of haematoma.

**Figure 1:**
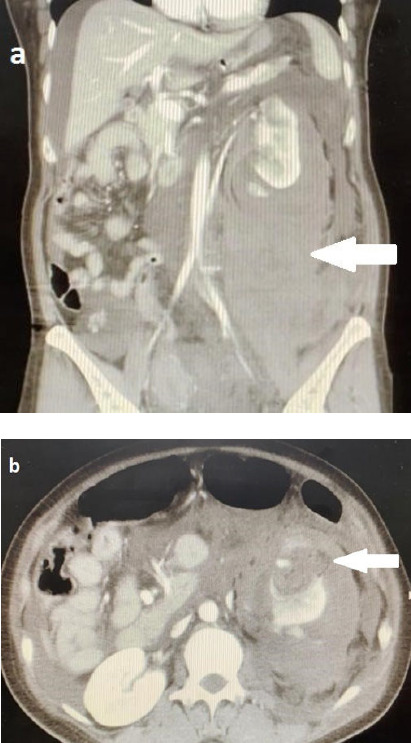
(a) Coronal CT image showing massive retroperitoneal haemorrhage mostly on the left side (arrow). (b) Axial CT image in the arterial phase showing a heterogeneous, mainly hypodense, mass at the lower pole of the left kidney suggestive of AML (arrow). AML, angiomyolipoma; CT, computed tomography

In the intensive care unit, the patient was in hypovolemic shock requiring inotropic support. CT revealed a large left retroperitoneal haemorrhage extending to the pelvis. There was a mass measuring 3.1×4.3×4.4 cm at the lower pole of the left kidney. There was evidence of rupture with active bleeding at the inferior part of the mass (Figure 1). The right kidney and other intraabdominal organs were normal. No lung cyst was seen.

Due to the life-threatening condition, the urology team decided to perform relaparotomy and urgent nephrectomy. There was an estimated 5-L blood loss during surgery. Histopathological examination confirmed the mass as AML of the typical (triphasic) type with positive results for vimentin, SMA, Melan-A and HMB-45 (image not available). After surgery, the patient gradually recovered and was discharged home after a few days.

## Discussion

Benign renal lesions are relatively rare, constituting 11%–17% of all renal lesions. Among these lesions, AML is the most frequently encountered type, accounting for 13%–52% of all benign renal lesions.^1^ It affects 0.2%–0.6% of the total population worldwide, with women accounting for 78% of cases.^2^ The mean diagnostic age for AML during pregnancy is 31.4 years, whereas the mean gestational diagnostic age is 27.7 weeks.^3^

AML may occur sporadically or be associated with genetics. The sporadic pattern occurs in 80% of cases, while the remaining 20% are linked to tuberous sclerosis complex (TSC) or, less commonly, sporadic lymphangioleiomyomatosis (LAM).

Generally, >80% of patients with AML are asymptomatic, while rupture occurs in only 15% of cases.^2^ Conversely, almost 81% of AML cases during pregnancy rupture.^3^ Pregnant patients with ruptured AML commonly present to the emergency department with abdominal pain (76.2%) and shock (42.1%).^7^ The major differential diagnoses for such presentation are usually abruptio placenta and ectopic pregnancy.

The risk factors for AML-related haemorrhage include pregnancy, a tumour size of >4 cm, the presence of an aneurysm sized >5 mm, hormonal therapy, trauma and association with TSC/LAM. However, the tumour and aneurysm sizes do not directly influence the incidence of rupture, although the aneurysm size has better specificity in predicting future rupture than the tumour size.^5^

Pregnancy is an established risk factor for AML rupture. This is mainly attributed to its hormonal effect, which causes the tumour to grow faster and become more aggressive. This subsequently causes retroperitoneal haemorrhage, maternal shock and foetal death. Some studies show that >25% of the tumour carries oestrogen and progesterone receptors.^4^ Another cause of the increased tumour size during pregnancy is hydronephrosis due to relaxation of the ureters and gravid uterus. Increased blood volume and blood pressure during pregnancy alter the haemodynamics and contribute to aneurysm formation in the tumour. During labour, increased intra-abdominal pressure, haemodynamic disturbance and increased muscle sensitivity to oxytocin make AML more prone to rupture.^5^

CT and magnetic resonance imaging are helpful in diagnosing cases. The radiological appearance of AML varies, ranging from a typical fat-rich lesion (-10 Hounsfield units on unenhanced CT) to a fat-poor and fat-invisible lesion. Fat-poor AML may resemble renal cell carcinoma (RCC). Lesions that contain calcification favour the diagnosis of RCC, whereas the presence of fat favours the diagnosis of AML.^2^

Since there is no consensus on how to manage AML during pregnancy, treatment strategies are usually based on several factors, including maternal and foetal conditions, gestational age, availability of facilities, patients’ decision regarding continuation of pregnancy and association with TSC/LAM. Termination of pregnancy may be advised in patients who are diagnosed during early pregnancy.^5^ Many centres opt for caesarean section for full-term pregnancies, although this approach is not frequently associated with less risk of rupture. Conversely, vacuum delivery may help by shortening the second stage of labour.^3^

In emergency situations, embolisation is currently the first-line treatment method for patients with acute haemorrhage and haemodynamic instability.^3,5^ Embolisation has a high success rate of up to 90%.^6^ Surgery is a second option after embolisation and is reserved only for patients with failed embolisation in acute emergencies and those with severe complications of embolisation and life-threatening conditions. In emergency settings, nephrectomy can be life-saving.^6^

Due to the high risk of rupture, some authors suggest prophylaxis treatment for patients with AML sized >4 cm and AML associated with TSC/LAM even if they are asymptomatic. Prophylaxis embolisation can be recommended, which can be safely performed after 12 weeks of gestation with minimal radiation exposure to the foetus. Embolisation is also helpful in reducing the tumour size prior to surgery and, hence, its complications. Prophylactic nephrectomy (partial or total) is reserved for patients with substantially large AML, malig-nant transformation, failed embolisation and contraindication to embolisation.^6^ Nephrectomy may be an option in full-term pregnancies in combination with caesarean section.^5^ Surgery has an advantage of histologic confirmation of the tumour. However, surgery during mid-pregnancy is associated with poor foetal outcomes.^6^

A conservative approach may be undertaken for asymptomatic pregnant patients only if they are keen on continuing their pregnancy following counselling on the high risk of AML rupture. In these patients, definitive treatment is carried out after delivery. Conservative management is also recommended for AML sized <4 cm or for a larger but stable tumour.^5^ However, there is no recommendation on how long conservative management should be carried out.

Pre-pregnancy counselling on contraception should be offered to patients with known AML. The risk of rupture during pregnancy should be explained, and if patients are nevertheless keen to get pregnant, they must be informed about the treatment options and their risks in case their AML ruptures.

Mechanistic target of rapamycin inhibitors (e.g. everolimus) are offered to non-pregnant patients with both TSC/LAM and AML who are asymptomatic with lesions sized >3 cm. However, these drugs belong to category C, with no adequate studies on their safety.^4^ Other treatment options that are offered to nonpregnant patients but have not been studied in pregnant patients include cryoabla-tion and percutaneous radiofrequency ablation. These techniques are reserved for patients with small and asymptomatic lesions.

## Conclusion

AML is a rare benign lesion that can present with severe life-threatening complications. Pregnancy is a major risk factor for rupture and massive haemorrhage owing to its hormonal effect. The possibility of AML rupture should be considered when encountering pregnant patients with hypovolemic shock but with normal initial obstetric ultrasound findings and ruled out abruptio placenta and ectopic pregnancy. The treatment options are individualised based on many factors. Embolisation and nephrectomy are life-saving treatments for patients with acute haemorrhage. Further, multidisciplinary management is recommended.
